# Beyond glucose and Warburg: finding the sweet spot in cancer metabolism models

**DOI:** 10.1038/s44324-024-00017-2

**Published:** 2024-09-02

**Authors:** Nia G. Hammond, Robert B. Cameron, Brandon Faubert

**Affiliations:** Department of Medicine, Section of Hematology/Oncology, University of Chicago, Chicago, IL USA

**Keywords:** Cancer metabolism, Mitochondria, Cancer

## Abstract

Advances in cancer biology have highlighted metabolic reprogramming as an essential aspect of tumorigenesis and progression. However, recent efforts to study tumor metabolism in vivo have identified some disconnects between in vitro and in vivo biology. This is due, at least in part, to the simplified nature of cell culture models and highlights a growing need to utilize more physiologically relevant approaches to more accurately assess tumor metabolism. In this review, we outline the evolution of our understanding of cancer metabolism and discuss some discrepancies between in vitro and in vivo conditions. We describe how the development of physiological media, in combination with advanced culturing methods, can bridge the gap between in vitro and in vivo metabolism.

## Introduction

Altered metabolism is a pivotal feature of cancer cells. Cancer cells reprogram metabolic pathways to support dysregulated cell growth and proliferation compared to non-malignant cells. Examples of altered metabolic pathways in cancer include reprogrammed aerobic glycolysis, glutamine catabolism, redox homeostasis, and numerous biosynthetic processes, all of which support the energetic and biosynthetic demands of deregulated cell growth^1,2^. Metabolic rewiring can support other hallmarks of cancer, such as proliferative capacity, immune response, and metastasis^3–5^. Thus, understanding metabolic reprogramming in cancer can lead to important insights into the underlying pathophysiology of this disease.

The origins of metabolic alterations in cancer can be traced back over a century. In 1924, Otto Warburg made a discovery that has now become synonymous with the modern concept of metabolic reprogramming in cancer (several excellent reviews detailing his work are available^6–9^). Warburg’s seminal observation, now referred to as the “Warburg Effect”, was that cultured tumor tissues displayed high rates of glucose uptake and lactate secretion, even in the presence of adequate oxygen. This starkly contrasts the metabolism of the non-malignant cells studied by Warburg’s contemporaries Herbert Crabtree and Louis Pasteur. In non-malignant cells, Crabtree and Pasteur observed a metabolic balance between glycolysis and oxidative phosphorylation (OXPHOS). Increasing glucose levels could impair OXPHOS, while inversely, high oxygen levels could impair glycolysis^10,11^. Thus, an important aspect of the Warburg Effect is the disproportion between glycolysis and respiration. To this day there are still misconceptions about Warburg’s work, or more specifically, Warburg’s interpretation of the data^12^. Warburg hypothesized that tumors have an “irreversible injuring of respiration” and that this was a central cause of cancer^13^. Debate over this interpretation was fierce, driven in part by Warburg’s penchant for antagonistically criticizing those who disagreed with him^14–16^. Despite his interpretation, Warburg’s own data indicated that cancer cells indeed respire, and have been confirmed in several subsequent works^10,17,18^.

**Fig. 1:**
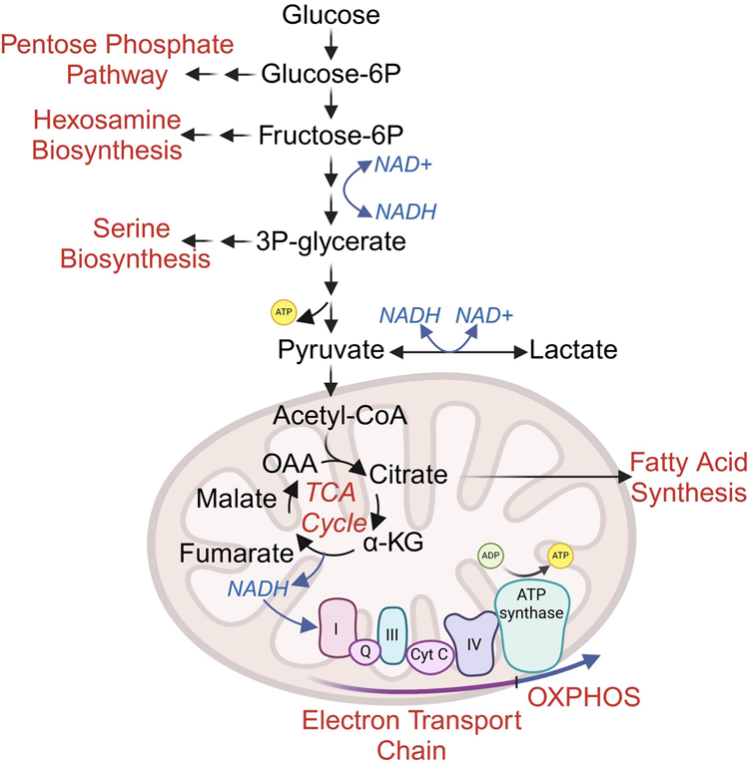
Central carbon metabolism fuels energy and biosynthesis. Glucose metabolism fuels several biosynthetic and energy-generating pathways (red). Glycolytic intermediates can be re-directed from glycolysis to other biosynthetic pathways, supporting DNA and RNA synthesis (pentose phosphate pathway), glycan generation (hexosamine biosynthesis), or one-carbon metabolism (serine biosynthesis). Pyruvate entry to the TCA cycle results in both high levels of ATP generation and biosynthesis pathways such as fatty acid synthesis. OAA oxaloacetate, α-KG alpha-ketoglutarate.

Mitochondria play critical roles as an energetic and biosynthetic hub in cancer. Pyruvate entry into the mitochondria fuels the tricarboxylic acid (TCA) cycle, a series of enzymatic reactions that generate reduced electron carriers that enter the electron transport chain and generate energy in the form of ATP. Mitochondria also generate numerous biosynthetic intermediates^19^ and contribute to intracellular signaling via the generation of reactive oxygen species^20^, all of which play key roles in supporting the growth and proliferation of cancer cells. Yet, questions about the function and necessity of mitochondria in cancer persist. For instance, why do tumor cells use glycolysis, a pathway that produces less ATP to support proliferation? Several noteworthy investigations offer some insight. First, an advantage of glycolytic metabolism is its speed, estimated to be 10–100× the rate of oxidative phosphorylation^21^. Second, glucose can support several biosynthetic pathways, including the pentose phosphate pathway, producing the sugar backbones for DNA and RNA; hexosamine biosynthesis, producing uridine diphosphate N-acetyl glucosamine, which is used for glycosylation; or serine biosynthesis, which supports one-carbon metabolism, fatty acid metabolism, and other processes^7^. Thus, a key advantage of glycolytic reprogramming in cancer is increased biosynthetic capacity (Fig. 1).

It is a testament to the importance of Warburg’s discovery that cancer metabolism remains a key area of interest. His findings have inspired generations of scientists to investigate how and why metabolism is reprogrammed in cancer^22^. It is now appreciated that cancer metabolism is highly heterogeneous and adaptable, with distinct metabolic features between and within cancer types^23^. Nutrient use, enzyme activity, and overall metabolic pathway engagement can be significantly altered depending on the model system and growth conditions used to investigate the cancer cells. This review highlights recent advances in metabolic reprogramming in cancer, focusing on bridging the gap between in vivo and in vitro studies of cancer metabolism.

## Methods of measuring tumor metabolism

**Fig. 2:**
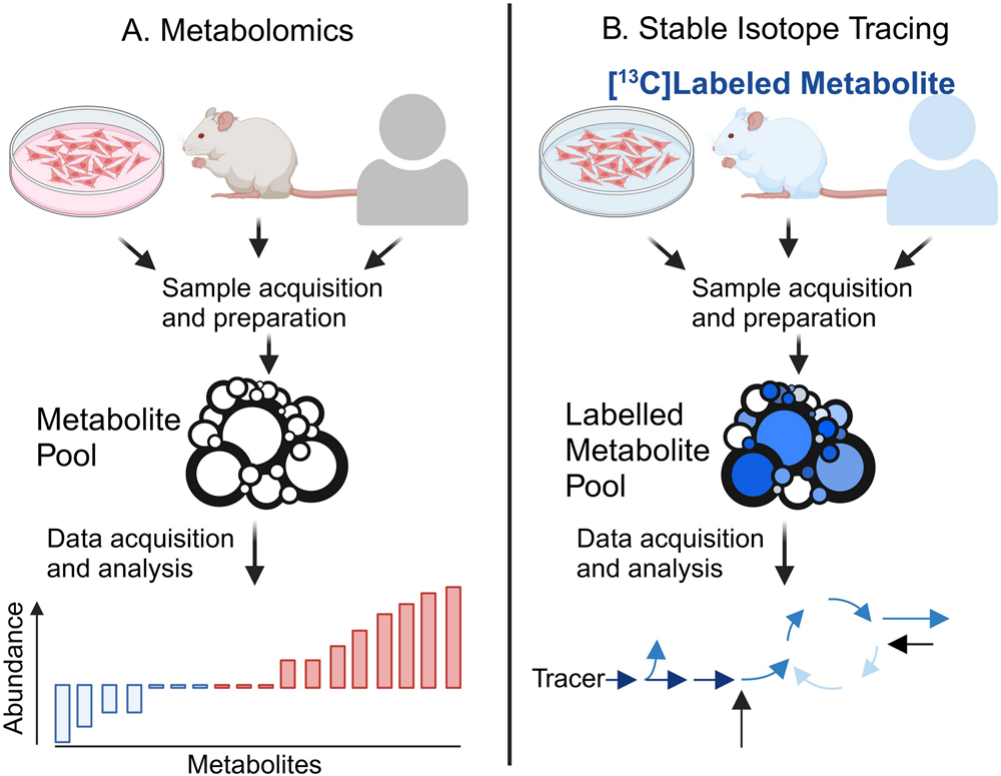
Utility and readouts of metabolomics and stable isotope tracing. **A** In metabolomics, samples are obtained and processed using mass spectrometry to identify the abundance of various metabolites. As such, individual metabolites are readily identified, and differences in the sizes of different metabolite pools can be identified (shown here by the size of various circles). While more sophisticated analyses can be used to model the activity of various metabolic pathways, metabolomic studies cannot directly assess pathway activity. **B** Stable isotope tracing requires the addition of a mass-labeled metabolite (e.g., ^13^C-glucose,^15^-N-glutamine) to the system (represented here by blue overlays of various models). After a set incubation period, samples are obtained and processed for mass spectrometry. This technique provides a greater depth of information regarding the utilization of different nutrients and metabolites by incorporating labeled atoms into different metabolites (represented here by blue shading of circles). Thus, pathway activity is directly assessed.

Measuring tumor metabolism generally relies on two broad techniques: metabolomics, which analyzes hundreds of metabolites at a given time point, or stable isotope tracing, which provides insight into how a nutrient is metabolized in different pathways (Fig. 2). These techniques offer complementary insights into metabolic phenotypes of tumors (several detailed reviews are available^24–26^). In brief, the measurement of metabolites is most commonly performed using nuclear magnetic resonance spectrometry (NMR) or mass spectroscopy (MS), and samples from both metabolomics and isotope tracing can be quantified on these platforms.

Metabolomics is an informative technique for profiling a broad array of metabolic features. These sensitive methods detect hundreds of metabolites, reporting on absolute or relative abundances. This technique is commonly applied in biomarker discovery and tumor characterization, which correlates metabolite abundances with tumor type, mutation, size, or patient outcome^27,28^. For example, mutations in metabolic enzymes such as isocitrate dehydrogenase (IDH), a TCA cycle enzyme, occur in cancers such as glioma^29^ and acute myeloid leukemia^30^. This mutation produces a neomorphic metabolite R-2-hydroxyglutarate, which can be distinguished from the non-mutated IDH product α-ketoglutarate, providing a diagnostic characteristic to identify this mutation and highlights the utility of metabolomics as an investigational and clinical tool.

While metabolomics identifies differences in metabolite abundance, it is difficult to resolve pathway activity or the flow of metabolites through a system using this method alone. Stable isotope tracing is a complementary approach to address these gaps. Stable isotopes (e.g., non-radioactive) are elements that contain an extra neutron and can be distinguished from endogenous isotopes by nuclear magnetic resonance or mass spectrometry. A nutrient labeled with a stable isotope (e.g., [U-^13^C]glucose) is added to a biological system (cultured cells, animal models, or patients). As the labeled nutrient is metabolized to downstream products, the ^13^C label is transferred and the position and extent of labeling provide information on enzymatic activity. The choice of labeled nutrient and label location allow for investigations into nutrient utilization, metabolic fate, and potential enzymatic activity within a system. By employing multiple tracers across a series of time points, metabolic flux analysis can be performed to provide quantitative evidence of how metabolic pathways operate within a given biological system^31^.

## Analyzing metabolism in vivo: lessons from stable isotope tracing studies

Our understanding of metabolic reprogramming in cancer has accelerated over the past decade. The use of metabolomics, stable isotope tracing, and metabolic flux analyses on tumors in vivo^24^, including directly measuring tumor metabolism in patients with cancer^32^, provides important information on nutrient supply and relative metabolic activity within tumors. Stable isotope infusions into patients at the time of a biopsy or resection generate important insights into bona fide human tumor metabolism, from which further studies could be based.

An early goal of these tracing studies was to identify which nutrients contribute to tumor metabolism in vivo and how tumor metabolism compares to adjacent benign tissue. Collectively, the largest cohort to date is patients with non-small lung cancer (NSCLC) who were infused with [^13^C]glucose. These studies demonstrated that ^13^C enrichment in central carbon metabolites (e.g., glycolysis and the TCA cycle) was very heterogeneous, but overall enrichment was far higher in tumors than adjacent lung^33–36^. In fact, nearly all tumor types studied have had significant ^13^C incorporation into glucose and TCA cycle metabolites, including glioma and brain metastases^37^, pediatric tumors of multiple histologies^38^, and breast cancer^39^. The lone outlier (to date) of this phenotype is clear cell renal cell carcinoma (ccRCC), which displays minimal contribution of glucose to the TCA cycle^40^. This lack of glucose contribution to the TCA cycle may be due to genetic perturbations in ccRCC, as approximately 90% have inactivation of the von Hippel Lindau (VHL) ubiquitination complex, leading to stabilization of HIFα subunits that enhance glycolysis and decrease pyruvate metabolism in the mitochondria^41^. A non-mutually exclusive possibility is the hypoxic nature of the kidney, as ccRCC metastases in the lung increase glucose contribution to the TCA cycle^42^. Animal models also offer important insights into tumor metabolism. An added advantage of these model systems is the capacity to measure ^13^C tissue enrichment at multiple time points, providing for a more detailed analysis of metabolic flux. One intriguing evaluation of TCA cycle metabolism in a series of cancer types found that flux depends on both tumor type and location. Solid Kras-driven tumors exhibited lower TCA flux than healthy tissues, whereas flux was higher in hematogenous cancers^43^. Interestingly, metastatic breast cancers displayed increased TCA cycle flux relative to primary tumors^43^, supporting the notion that tumor metabolism is altered during metastatic progression and is influenced by the metastatic site^44,45^.

Studies of tumor metabolism in vivo have revealed some disconnects with the metabolic phenotypes of cultured cells. For example, in mouse models of Kras-driven NSCLC, cultured cells catabolize glutamine to support the TCA cycle, whereas this contribution is negligible for NSCLC in vivo^46^. Importantly, these metabolic differences also affect gene essentiality for cancer cell growth. Genetic deletion of glutaminase (GLS) results in cell death of cultured NSCLC, but these same cells are agnostic to GLS loss in vivo^46^. Later studies revealed that the reliance on glutamine catabolism in cultured cells is at least partially due to the media composition, which contains artificially high levels of cystine. Depleting cystine levels in vitro reduces the overall contribution of glutamine to the TCA cycle in cultured cells^47^. Similar discordance between cultured and in vivo systems has been observed in models of pancreatic cancer. Knockout of the mitochondrial isoform of glutamate-oxalate transaminase (GOT2) impairs pancreatic cancer growth in vitro, but growth is unaffected in vivo^48^. In separate studies of pancreatic cancer, knockout of N-acetylglucosamine kinase (NAGK) had no appreciable effect on cell growth under nutrient-replete culture conditions, but renders these cells unable to grow in a mouse model^49,50^. Overall, these studies demonstrate potential disconnects between model systems that can have important implications for our understanding of tumor metabolism, and highlight the need to consider how we investigate metabolism in these systems carefully.

## Modeling tumor metabolism in cultured cells with 2D and 3D systems

Tumor metabolism is affected by both intrinsic factors (e.g., genetic mutations) and extrinsic factors within the tumor microenvironment (TME). While the cancer cells themselves may be highly heterogeneous both genetically and metabolically, the TME is a complex mixture of different cell types, including stromal, immune, and non-malignant epithelial cells. Further adding to this complexity, tumors can be influenced by variations in vasculature and mechanical forces^51^. Thus, while tumor metabolism in vivo is impacted by a complex combination of intrinsic and extrinsic factors, standard culturing methods can only partially replicate these conditions^52^.

Two-dimensional (2D) tissue culture is the conventional approach to studying many cancer cell types. Cells are typically grown in a monolayer and generally only include a homogeneous population of cancer cells. First, despite this relative simplicity and lack of extrinsic factors, 2D culture methods can provide good fidelity to in vivo models. Metabolic CRISPR screens demonstrated that 2D culture recapitulates the majority (~85%) of dependencies of pancreatic cancer cells identified in vivo^53,54^. Yet, differences of gene essentiality between these model systems were still present; for instance, cultured cells failed to predict the in vivo dependency on heme metabolism. In the cultured version of the CRISPR screen, cells that were capable of synthesizing heme could export a biosynthetic precursor that was metabolized by neighboring cells to compensate for *Hmbs* loss^53,54^. These studies revealed an important, cancer-type-specific aspect of gene essentiality. When the top-performing sgRNA for both in vitro and in vivo Kras-PDAC models were applied to Kras-driven NSCLC, only a few shared metabolic essentialities were observed^54^.

Cellular complexity can be increased with spheroid culture systems, wherein cells form 3-dimensional aggregates in suspension. More recently, organoid models, in which cells are suspended in an extracellular matrix such as Matrigel, have been developed to model the interactions between the tumor and its surrounding matrix as discussed in depth in the following review^55^. Different groups have attempted to identify metabolic similarities and differences across 2D culture, 3D culture, and in vivo systems. In the above studies of metabolic CRISPR screens in PDAC, the authors observed metabolic differences between 2D and 3D culture models, with the 3D culture models providing greater fidelity to in vivo tumor growth^53,54^. In some cases, these metabolic differences can better model patient outcomes. In a study of patient-derived PDAC organoids, metabolic profiling was able to stratify patients into two subtypes that were associated with worse patient outcomes^56^. More aggressive tumors were associated with reprogrammed glucose metabolism through the GLUT1/ALDOB/G6PD axis, leading to increased glucose utilization via glycolysis and the pentose phosphate pathway. Ultimately, this promoted chemoresistance by increasing pyrimidine synthesis^56^. Targeting this axis sensitized tumors to cytotoxic chemotherapy, suggesting that this pathway may represent a therapeutic target for PDAC. Three-dimensional cultures also improve fidelity to in vivo models of breast cancer. Here, 3D culture led to a higher expression of proline dehydrogenase than in 2D culture, providing a better representation of patient data, where metastases have increased proline dehydrogenase expression^57^. This difference in expression leads to increased proline catabolism to support ATP production, and inhibition of proline dehydrogenase decreased metastases in mouse models^57^.

Advances in 2D and 3D culture models provide a greater capacity to discover metabolic features that are key to tumor biology in vivo. For instance, 3D systems are being used to model regional differences in metabolism including drug sensitivity^58^, and advances in bio-printing and co-culture systems can be used to model tumor and immune cell interactions^59^. Ultimately, the choice of culture system depends on the experimental question being posed. Interrogating metabolism with 2D culture can be sufficient from an experimental perspective in many aspects, but can be difficult to extrapolate to more complicated systems in others. Pushing the capacity of these models to mirror in vivo biology requires multiple investigational approaches, and one important consideration is using culture media that more accurately represents the in vivo nutrient environment of the tumor.

## Mimicking the in vivo nutrient environment with physiological media

**Fig. 3:**
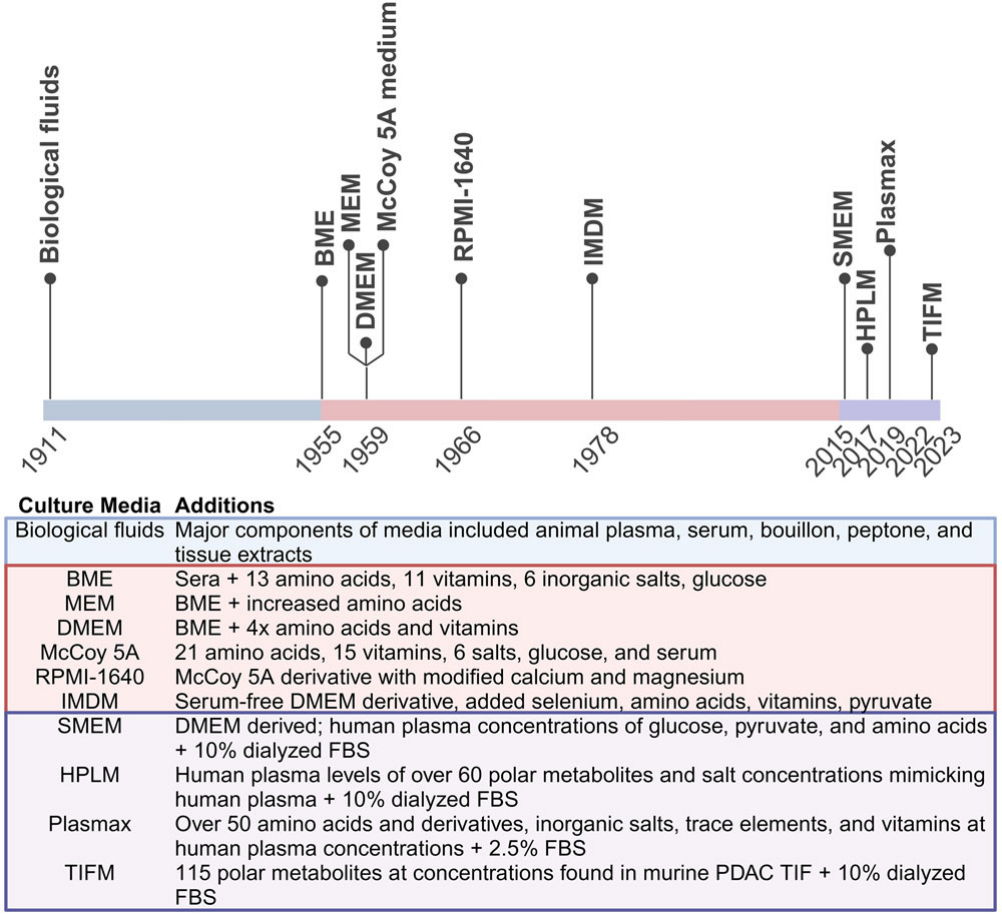
Timeline of tissue culture media development and formulation. In the early 1900s, biological fluids such as plasma were common practice for culturing cells. This natural media period continued until the development of BME in 1955. This was shortly followed by the rapid generation of other synthetic media designed for different cell types that have now become standard for in vitro work today. However, a recent physiologic media revolution initiated in 2015 with SMEM has ushered in a new wave of media development modeling in vivo environments.

Standard media such as DMEM and RPMI-1640 (RPMI) were developed in the mid-20th century to maximize cellular proliferation with the minimum number of nutrients, while minimizing the need to change media^60–63^. As such, these media contain supra-physiological levels of nutrients such as glucose and glutamine, while multiple nutrients deemed unimportant or unnecessary for proliferation are absent. In contrast, physiological media have been developed over the last decade to contain physiological concentrations of metabolites found in various in vivo environments such as serum or tumors, to varying degrees of complexity. For in-depth reviews of the history of media and the recent development of physiologic media, see the following reviews^64–66^ (Fig. 3). We briefly describe their formulations here.

Serum-like tissue culture medium (SMEM) was one of the early physiological media developed^67,68^. It was designed by modifying DMEM to contain amino acids, glucose, and pyruvate at concentrations found within healthy human serum. More systematic approaches have been used in the design of recent physiological media. For example, human plasma-like medium (HPLM) was generated to contain a series of amino acids, polar metabolites, and small ions at concentrations found in healthy human plasma, according to the Human Metabolome Database^69^. The development of Plasmax involved a similar approach, with the additional inclusion of amino acid derivatives and other trace elements^70^. Additionally, the amount and type (e.g., unmodified vs. dialyzed) of fetal bovine serum (FBS) are also important considerations and differ across these formulations, with HPLM utilizing 10% dialyzed FBS and Plasmax requiring 2.5% FBS.

While these media provide a more accurate approximation of the serum or plasma nutrient environment, the TME can be metabolically distinct from serum^71,72^. In vivo, cancer cells are surrounded by interstitial fluid (tumor interstitial fluid, or TIF), representing the closest approximation of the nutrients available to cancer cells^73^. By quantifying the metabolite concentration of isolated tumor interstitial fluid, Tumor Interstitial Fluid Medium (TIFM) was developed to contain 115 polar metabolites at concentrations measured in the interstitial fluid of murine pancreatic ductal adenocarcinoma (PDAC) tumors^74,75^. As with previously developed media, the metabolites included in this formulation were based on commercial availability and stability. Defining the TIF nutrient concentrations in specific tumor types, or the interstitial nutrient metabolome of healthy tissues, are active areas of investigation.

## Biological insights from growing cancer cells in physiological media

Using a media that mimics both the concentration and availability of metabolites in vivo can significantly alter the metabolism of cultured cancer cells. Several studies have demonstrated that culturing cancer cells in physiological media can better recapitulate the metabolic phenotype of tumors in vivo, and have observed changes in glutamine, arginine, nucleotide, and mitochondrial metabolism.

Recent work has documented alterations in glutamine metabolism when culturing cells in physiological media. As discussed above, glutamine anaplerosis can be influenced by the media formulation^47^, and similar metabolic changes were observed in glioblastoma cells cultured in SMEM. Here, glutamine did not enter the TCA cycle, but instead, these cells re-directed glutamine towards purine synthesis, mimicking the phenotypes in both patients with glioblastoma and in xenograft models^68^. Similarly, culturing human blood cancer cell lines in HPLM reduced the essentiality of glutaminase compared to RPMI in CRISPR screens. The difference in gene essentiality was partially due to the absence of pyruvate in RPMI, but its presence in HPLM^76^. Similar results were also observed in glioma cells cultured in Plasmax^77^.

Arginine metabolism is also significantly impacted by nutrient levels in the media. Culturing PDAC cells in TIFM causes arginine synthesis via argininosuccinate synthase 1 (ASS1), consistent with in vivo studies, yet is not observed in PDAC cells cultured in RPMI. This disparity is due to the low levels of arginine present within PDAC TIF, approximately 2–5 μM compared to the 125 μM found in plasma and 1.15 mM in RPMI^74^. Similar effects have been observed in breast cancer cells, where the artificially high arginine levels in DMEM F-12 result in the enzymatic reversal of argininosuccinate lyase (ASL), which canonically catalyzes the conversion of argininosuccinate to arginine and fumarate. However, this directionality was restored in cells cultured in Plasmax^70^. Furthermore, the reduced levels of arginine present in Plasmax induced de novo serine synthesis and elevated ATF4 expression in Plasmax-cultured cancer cell lines, suggesting broader metabolic rewiring beyond arginine synthesis^78^.

Physiological media has also provided novel insights into nucleotide metabolism that would have been missed using standard media formulations. Culturing hematological cancer cell lines in HPLM impairs pyrimidine synthesis^69^. This effect is mediated by uric acid, which is present in human plasma but is largely absent from RPMI. Uric acid directly inhibits the OMP decarboxylase (ODC) domain of UMP synthase (UMPS), and a consequence of this inhibition is a reduced sensitivity to the antimetabolite chemotherapeutic 5-fluorouracil (5-FU). Physiological media may also alter the choice of carbon source for nucleotide synthesis. Plasmax cultured cancer cell lines have an elevated contribution of hypoxanthine salvage and de novo serine synthesis-derived carbons to fuel purine synthesis compared to DMEM cultured cells^78^. In DMEM cultured cells, exogenous hypoxanthine is absent, and extracellular serine import, instead of de novo serine synthesis, contributes to purine metabolism. Because of this, combined inhibition of both pathways had an additive effect in reducing proliferation in Plasmax cultured cells and may provide a potential therapeutic strategy^78^.

Lastly, several groups have investigated the impact of physiological media on mitochondrial metabolism. In one example, the supraphysiological levels of pyruvate in DMEM-F12 induced a hypoxic signature through the stabilization of HIF1α in breast cancer cells, which was reversed when cells were cultured in Plasmax^70^. Investigations into the influence of medium choice on mitochondria reveal that physiological media alterations in mitochondrial respiration and morphology characteristics have been largely cell-line specific, as no consistent trends have emerged^69,79–81^.

## Important considerations for media, models, and metabolism

While physiological nutrient conditions can better mimic some aspects of metabolism, the reduced concentrations of many nutrients make these experiments more prone to depletion and nutrient stress. Notably, even when cancer cells are cultured with excess media, 48 h without media change severely depleted glucose and the majority of amino acids in Plasmax^82^. This leads to an enrichment in nutrient stress response pathway signatures by RNA-sequencing. Media changes every 24 h mitigate this nutrient depletion and stress response signature; however, 24 h of culture still depleted glucose and a handful of amino acids below the healthy human plasma range. Thus, efforts should be made to avoid dropping nutrient levels below normal physiological concentrations by performing frequent media changes or using continuous flow culture systems^83^. Secondly, the type of FBS added to media should be considered. The inclusion of dialyzed or unmodified FBS significantly impacts gene essentiality in CRISPR screens^76^. Dialyzed FBS versus unmodified FBS impacted the hits identified in RPMI and DMEM cultured cells. This is because FBS contains a mix of unknown metabolites and lipids that can vary^65^. Therefore, thoughtful consideration of the FBS supplement and percentage should be included in the experimental design.

Not all metabolic phenotypes require the use of physiological conditions to match in vivo observations, as highlighted by metabolic CRISPR screens for gene essentially^53,54^. Furthermore, simply altering the levels of specific metabolites can provide useful insights into biological responses. To determine how PDAC cells might behave in media conditions more similar to the in vivo environment, reduced nutrient levels were modeled by altering standard DMEM to contain 2.5 mmol/L glucose, 0.5% FBS, and one-fifth of the standard amino acid concentration. Here, PDAC cells were found to mimic the poorly perfused regions of pancreatic tumors more accurately^84^. Relatedly, glucose concentrations within multiple tumors are commonly several-fold lower than in non-transformed tissue^85,86^. By culturing cells at a lower but constantly maintained glucose level, Birsoy et al. observed significant metabolic diversity and sensitivity to OXPHOS inhibition across cell lines that were otherwise masked in standard cell culture conditions^83^.

All model systems come with significant limitations. Cultured cells are a population of highly homogeneous cells, in contrast to tumors in vivo, wherein cancer cells interact with fibroblasts, endothelial cells, immune cells, and contain various cancer cell subclones within a tumor. Metabolic interactions with these different cell populations plays an important role in the tumor metabolic phenotype. Isotope tracing studies in mice bearing PDAC tumors identified that some tumor metabolic patterns were only induced by the presence of cancer-associated fibroblasts, both in vivo and co-culture systems in vitro^87^. The concentration of most key metabolites can be tightly regulated in vivo, even with significant fluctuations in metabolite availability from dietary intake^75^. In contrast, in vitro systems lack these homeostatic mechanisms and can be subject to substantial temporal changes in nutrients. The use of bioreactor systems where the media can be constantly replenished^83^, may offer unique insights into the homeostatic mechanisms of cultured cancer cells. One key limitation of measuring metabolism in vivo is the difficulty in resolving cell-type specific metabolism with current technological methods. Standard sample processing for metabolomics and stable isotope tracing involves homogenizing tissue samples before analysis, resulting in the mixture of metabolites from multiple cell types. The application of spatial metabolomics can be used to identify metabolites in situ, allowing for the resolution of metabolic differences between cell types or areas^88^. While the use and advances in these platforms are rapidly accelerating, several technical hurdles (image resolution, sensitivity, imaging and processing speeds, etc.) are still being improved. Nevertheless, these techniques offer exciting future opportunities to investigate metabolic differences in situ.

## Conclusions and future directions

Tumor metabolism is a dynamic hallmark impacted by intrinsic and extrinsic factors, therefore, it is important to consider the influence of any model system on the metabolic phenotype. As reviewed here, one way to yield meaningful data is to alter cell culture conditions to mimic the physiology of the tumor ecosystem. Recent advances in culture media formulae, detection techniques, and interrogating metabolism in vivo, have allowed for more granularity in understanding cancer metabolism. As further improvements are implemented (Box 1), scientists and clinicians can better leverage tumor-specific metabolic derangements for patient benefit.

Box 1 Future questions and perspectives

- Advancing spatial techniques to better understand intratumoral heterogeneity and cell-cell crosstalk.
- Continuing development of additional physiologic media to better characterize the effects of different in vivo environments such as metastatic organs, diets, and tumor microenvironments.
- Investigating cancer metabolism directly in patients, expanding to additional cancer types, metastatic sites, and other stable isotope tracers.
